# Reducing Stress and Enhancing Academic Buoyancy among Adolescents Using a Brief Web-based Program Based on Acceptance and Commitment Therapy: A Randomized Controlled Trial

**DOI:** 10.1007/s10964-018-0973-8

**Published:** 2018-12-17

**Authors:** Anne Puolakanaho, Raimo Lappalainen, Päivi Lappalainen, Joona S. Muotka, Riikka Hirvonen, Kenneth M. Eklund, Timo P. S. Ahonen, Noona Kiuru

**Affiliations:** 1 Department of Psychology, University of Jyväskylä, P.O. Box 35, Jyväskylä, 40014 Finland; 2 Department of Psychology and Gerocenter, University of Jyväskylä, P.O. Box 35, Jyväskylä, 40014 Finland; 3 Faculty of Education and Psychology, University of Jyväskylä, P.O. Box 35, Jyväskylä, 40014 Finland

**Keywords:** Acceptance and commitment therapy, Stress, Academic buoyancy, Adolescents, Mobile intervention, Randomized controlled trial

## Abstract

Acceptance and commitment therapy programs have rarely been used as preventive tools for alleviating stress and enhancing coping skills among adolescents. This randomized controlled trial examined the efficacy of a novel Finnish web- and mobile-delivered five-week intervention program called Youth COMPASS among a general sample of ninth-grade adolescents (*n=* 249, 49% females). The intervention group showed a small but significant decrease in overall stress (between-group Cohen’s *d* = 0.22) and an increase in academic buoyancy (*d=* 0.27). Academic skills did not influence the intervention gains, but the intervention gains were largest among high-stressed participants. The results suggest that the acceptance and commitment based Youth COMPASS program may be well suited for promoting adolescents’ well-being in the school context.

## Introduction

Around 10–20% of adolescents in the West suffer from symptoms of stress, anxiety, and depression (Polanczyk et al. 2015; WHO 2014), which may hinder academic achievement (Cortiella and Horowitz 2014). However, individual factors, such as academic buoyancy, which refers to a student’s capacity to overcome everyday academic life setbacks and challenges successfully (Martin and Marsh 2009), may protect from the effects of psychological distress in the school context. New easily applicable methods are called upon to prevent and mitigate adolescents’ psychological distress and to support academic buoyancy, thereby potentially promoting adolescents’ subsequent mental health and successful educational careers. In addition, intervention methods should work equally well for adolescents with poor academic skills and those with normally developing skills.

There are a plethora of studies of adult populations showing that brief modern therapeutic methods and programs based on acceptance and commitment therapy are effective in the treatment of a variety of psychological conditions, including stress, depression, and anxiety (Flaxman et al. 2013; Hayes et al. 2012; Powers et al. 2009; Ruiz 2012). Moreover, a growing number of studies has used the acceptance and commitment approach in the treatment of adolescents, that is, 11–16-year-olds, for example, to alleviate depression (e.g., Petts et al. 2017), to alter behavioral outcomes (e.g., Armstrong et al. 2013), and to relieve physical conditions, such as pain (e.g., Wicksell et al. 2009). In general, acceptance and commitment approach based interventions for adolescents have shown positive effects in alleviating psychological symptoms, increasing quality of life, and enhancing psychological flexibility (Swain et al. 2015). However, studies in school settings are rare. Most of the previous studies have focused on clinical samples, that is, on participants who have significantly elevated psychological or physical symptoms or diagnosable disorders. In addition, in previous studies, the study designs and intervention programs have often been either poorly specified or included only some elements of the acceptance and commitment therapy protocol (Swain et al. 2015). Thus, due to previous research limitations, more information is needed on the usability of acceptance and commitment based interventions among adolescents in alleviating stress and promoting coping with academic setbacks in school settings.

Over the last few years, new web- and mobile-based acceptance and commitment interventions have emerged and have been used successfully among adult participants (e.g., Lappalainen et al.,2014a; Lappalainen et al. 2014b; Lappalainen et al. 2015; Puolakanaho et al. 2018) and university students (Räsänen et al. 2016). It is likely that mobile-based interventions are also suitable for adolescents who are familiar with using modern technology in their everyday lives. However, no previous studies exist in which web- or mobile-based acceptance and commitment programs have been used with adolescents to enhance their stress coping. In contrast, interventions have hitherto been carried out in face-to-face meetings either individually or in groups. Consequently, the aim of this study was to examine whether the novel web- and mobile-based acceptance and commitment program influenced stress symptoms and academic buoyancy in the school setting in a general (non-clinical) sample of ninth-grade adolescents. In addition, the study examined whether the efficacy of the acceptance and commitment intervention differs based on whether an adolescent has poor academic skills.

### Applying the Acceptance and Commitment Model to Adolescents

Acceptance and commitment therapy is described as a third-wave cognitive therapy developed by Hayes et al. (1999), and it focuses on recognizing participants’ own thoughts and emotions, as well as their connections to concrete behavior. More specifically, acceptance and commitment intervention models combine mindfulness and acceptance with behavioral principles and an understanding of personal values. *Mindfulness* refers to a state of consciousness in which attention is focused on present-moment phenomena, and *acceptance* refers to a willingness to experience all mental events (e.g., thoughts, emotions, and sensations) without changing, avoiding, or controlling them. *Behavioral principles* are used to clarify one’s personal values and to take actions that lead to goal accomplishment (Hayes et al. 2006; Hofmann and Asmundson 2008; Williams et al. 2008).

Recent acceptance and commitment intervention models have aimed to reinforce the six core psychological processes: the ability to remain flexibly and purposefully in the present moment and to be mindful of thoughts, feelings, bodily sensations, and action potentials; keeping a perspective-taking attitude on thinking and feeling; clarifying one’s hopes, values, and goals in life; doing and cultivating things in line with identified hopes, values, and goals; willingly accepting unwanted feelings by taking actions that are consistent with one’s hopes, values, and goals; and increasing defusion skills, i.e., observing and recognizing one’s thoughts that interfere with experienced life events and valued actions and seeing them as thoughts rather than literal truths (Flaxman et al. 2013; Hayes et al. 2012). Each of these processes is a psychological skill that can be enhanced in any life domain with regard to unwanted internal experiences or symptoms (e.g., thoughts, feelings, and physical sensations). Therefore, acceptance and commitment interventions are thought to be a trans-diagnostic psychological treatments that potentially influences multiple psychologically derived symptoms and life issues (Dindo et al. 2017; Hayes and Hofman 2017). These theoretical views also suggest that acceptance and commitment interventions may also work as preventive and early tools in alleviating diverse psychological symptoms (here, stress) and promoting well-being and health (here, academic buoyancy). The above-mentioned acceptance and commitment approach principles can also be used for planning interventions for adolescents. However, many factors ought to be considered when devising programs for adolescents (Ciarrochi et al. 2012; Halliburton and Cooper 2015; Hayes and Ciarrochi 2015; see also Steinberg 2002).

### Adolescent Stress and Acceptance and Commitment Interventions

Adolescents face multiple social, psychological, and physiological changes simultaneously in different life domains because of biological maturation, cognitive development, evolving sexuality, school transitions, and changes in social relationships, (e.g., Denham et al. 2009). In adolescence, conflicts with parents tend to increase and closeness with parents decreases as adolescents spend more time with their peers and come to value friendships more highly. Adolescents also face the challenges of learning to live independently and building their own social network by exploring and building their identities and starting romantic relationships (Steinberg and Morris 2001). At the same time, many adolescents experience stress in relation to school (e.g., Salmela-Aro et al. 2009; Seiffge-Krenke et al. 2012). Ninth-grade adolescents, who formed the target group of this study and who are finalizing their compulsory education, also face new personal and academic challenges because, for example, they have to complete a great deal of school projects and have to clarify their interests to be able to choose their future educational path. All these issues may elicit stress and may even lead to burnout (Salmela-Aro 2017) and other mental health-related problems, such as substance abuse and self-harm behaviors, anxiety, and depression (Avison 2010; Dyson and Renk 2006). It is also well known that stress experiences at younger ages are related to various psychological symptoms and clinical disorders in adulthood (Lee et al. 2014; Liu and Alloy 2010; Mundy et al. 2015). Thus, there is a need for effective strategies to protect youth from the dysfunctional effects of stress.

In an American survey, over 30% of teens reported being overwhelmed and depressed or sad due to stress (APA 2014). In a Swedish survey, 37% of girls and 22% of boys reported that they were frequently stressed (Wiklund et al. 2012). Similar trends have also been reported in a Finnish national school health survey (Finnish School Health Survey 2017), in which 32% of girls and 19% of boys reported serious schoolwork-related tiredness during the two last years of high school. Interestingly, the results of all these studies propose that girls are more likely than boys to experience stress (see also Wilhsson et al. 2016). These alarming figures call for novel and easily applicable methods to prevent and mitigate adolescents’ stress and to support their coping skills.

Some previous studies demonstrate that acceptance and commitment interventions can be effective in alleviating adolescents’ stress. For instance, Burckhardt et al. (2016) conducted an intervention study that included elements from acceptance and commitment therapy and positive psychology in a sample of high school students (*n=* 267, aged 16–17 years) in Australia. In their study, significant changes with medium-to-strong effect sizes were observed in the stress scores of students who commenced the program with high stress, depression, and anxiety scores. Livheim et al. (2015) conducted a pilot study in school settings of adolescents who were screened for psychological problems in Australia (*n=* 66) and Sweden (*n=* 32). In their study, significant improvements in large effect sizes were reported for stress. In his earlier study, Livheim (2004) conducted an acceptance and commitment program for youth (*n=* 230, aged 16–19 years), which revealed significant changes in stress and psychological flexibility that were also visible after two years. It is notable that the above-mentioned studies involved participants from clinical samples, that is, all the participants had elevated levels of psychological symptoms. Moreover, the interventions were implemented in face-to-face group settings and were led by trained counsellors. In contrast, no acceptance and commitment intervention results were found in a recent large-scale study conducted in school settings by teachers (Van der Gucht et al. 2017). Thus, although there is some evidence of the usefulness of acceptance and commitment interventions for adolescents, no previous study has used web or mobile technology to deliver the program individually to participants, which is the key topic of the current article.

### Adaptive Coping and Acceptance and Commitment Interventions

There are wide individual differences among adolescents in their experiences of stress, as well as in how they respond to or cope with stress (APA 2014). Building capacity for academic buoyancy can help them cope with challenges in academic life. Academic buoyancy refers to a positive and optimistic attitude toward everyday academic setbacks and an ability to deal with such setbacks in the course of ordinary life (e.g., poor performance, competing deadlines, performance pressure, difficult tasks; Martin and Marsh 2009). Academic buoyancy has been shown to be negatively associated with psychological risks, such as school or text anxiety, and with a lack of self-efficacy (Martin and Marsh 2008, 2009).

Some theoretical frameworks have combined stress management and the promotion of coping skills (e.g., Haase 2004), and these frameworks have been further tested in adolescent interventions, usually among clinical samples (e.g., Rosenberg et al. 2015). However, due to the trans-diagnostic nature of acceptance and commitment interventions and their proposed influence on deep psychological functions (e.g., Dindo et al. 2017; Hayes and Hofmann 2017), acceptance and commitment based exercises can be assumed to enhance adolescents’ self-awareness and independence. These can be further expected to increase their coping skills, such as academic buoyancy in the current study, against stressors, as well as to alleviate experiences of stress. A recent study (Hirvonen et al. 2018) found that high levels of academic buoyancy were associated with a lower level of stress later in school. As such, buoyancy can be assumed a kind of counterforce against stress, although it has not been studied in the acceptance and commitment therapy context among adolescents.

### Poor Academic Skills and Acceptance and Commitment Therapy

Poor academic performance may expose students to increased stress and psychological symptoms in comparison with better-performing students. A history of difficulties in learning and academic skills can lead, for example, to experiences of struggle, more conflicts with teachers and parents concerning homework, and increased negative emotions, such as frustration and disappointment in everyday learning situations (Cortiella and Horowitz 2014; Polanczyk et al. 2015). Consequently, it is possible that students with poor academic skills are particularly at risk for developing stress-related symptoms and could thus benefit from stress-reducing interventions. Yet, while some studies have noted that acceptance and commitment interventions may reduce psychological symptoms among students with academic challenges, there is little knowledge about whether acceptance and commitment interventions are similarly effective for students with different academic skill levels. Beauchemin et al. (2008) were able to improve the social and academic skills of adolescents with learning disabilities (n = 34) through five weeks of mindfulness exercises carried out by their teacher. Tentatively positive effects have also been shown in samples involving learning disabilities and anxiety (Brown and Hooper. 2009). Interestingly, no study has examined whether students’ academic skills moderate the efficacy of acceptance and commitment intervention in reducing stress and promoting academic buoyancy. This was one of the aims of the present study.

### Mobile and Web-based Interventions

A substantial amount of psychological interventions utilizing new digital technology has been developed over the past few decades. Their usage is not restricted to place and time and they are usually cost effective. In addition, they can be used without a specific therapeutic background, and they can provide new insights for different professionals, such as teachers. According to Andersson and Titov (2014), digital interventions are considered as efficient as face-to-face therapies, especially when they include certain features, such as motivation aspects and personal therapeutic support (see also Wozney et al. 2017).

Over the last few years, new web-based acceptance and commitment intervention programs have been developed among adults, which have been shown to be efficient in alleviating depression (Lappalainen et al. 2014a; Lappalainen et al. 2015) and work-related stress (Kinnunen et al. 2018) and in enhancing well-being (Lappalainen et al. 2014b). In a recent randomized controlled study, Räsänen et al. (2016) investigated the effects of a web-based acceptance and commitment program in a sample of university students (n = 68; ages 19–32 years) with varying levels of psychological distress. They found medium-to-large effect sizes in several well-being measures. However, there is no study of adolescents (aged 12–16 years) in which an acceptance and commitment intervention based program has been used and delivered utilizing web and mobile technology.

## The Current Study

The theoretical views presented above suggest that acceptance and commitment interventions may also work as preventive and early tools in alleviating diverse psychological symptoms (here, stress) and promoting well-being and health (here, academic buoyancy). In the current study, using the knowledge gained from new, complete acceptance and commitment intervention models for youth (e.g., Ciarrochi et al. 2012; Hayes and Chiarrochi 2015), a novel five-week web- and mobile-delivered intervention program called Youth COMPASS was developed. The main goal of the study was to explore the effects of the Youth COMPASS program on overall stress, school stress, and academic buoyancy among ninth-grade adolescents, who were in their last year of comprehensive school at the time of the study and therefore also vulnerable to stress-related experiences. The total sample of 249 adolescents was randomized into two acceptance and commitment intervention groups and a control group receiving only the usual support from the school. Half of the participants were identified as having poor academic skills.

The current randomized controlled trial study addressed three main research questions. First, to what extent can ninth-grade adolescents’ overall and school-related stress be reduced and academic buoyancy enhanced through the five-week web- and mobile-based acceptance and commitment intervention known as Youth COMPASS? Second, do the outcomes in the two intervention groups (which differed from each other slightly in the amount of personal face-to-face support) differ from each other regarding their efficacy, and do they differ from the control group’s outcomes? Third, do the adolescents’ poor academic skills moderate the efficacy of Youth COMPASS in reducing adolescents’ stress and enhancing their academic buoyancy? Based on the acceptance and commitment view, the intervention was thought to enhance underlying psychological processes and, through them, different well-being experiences. Consequently, it was expected that the level of stress will decrease and academic buoyancy will increase more in the two intervention groups than in the control group. Because there are no previous studies on the influences of poor academic skills on acceptance and commitment intervention outcomes among youth, no hypotheses were proposed for the last question.

## Methods

### Study Design and Randomization

The Youth COMPASS intervention study is part of the broader Stairway longitudinal research project, which aims to provide research-based knowledge of the individual- and environment-related factors that promote learning, well-being, and successful educational transitions. A subsample (*n* = 249) of students from the broader longitudinal study (*n*~800, here called the basic sample), carried out in two municipalities in Central Finland, were allocated for randomized controlled trials for the Youth COMPASS intervention. The interventions were carried out in the fall of the ninth grade (2017) before the transition to upper secondary school.

**Fig. 1:**
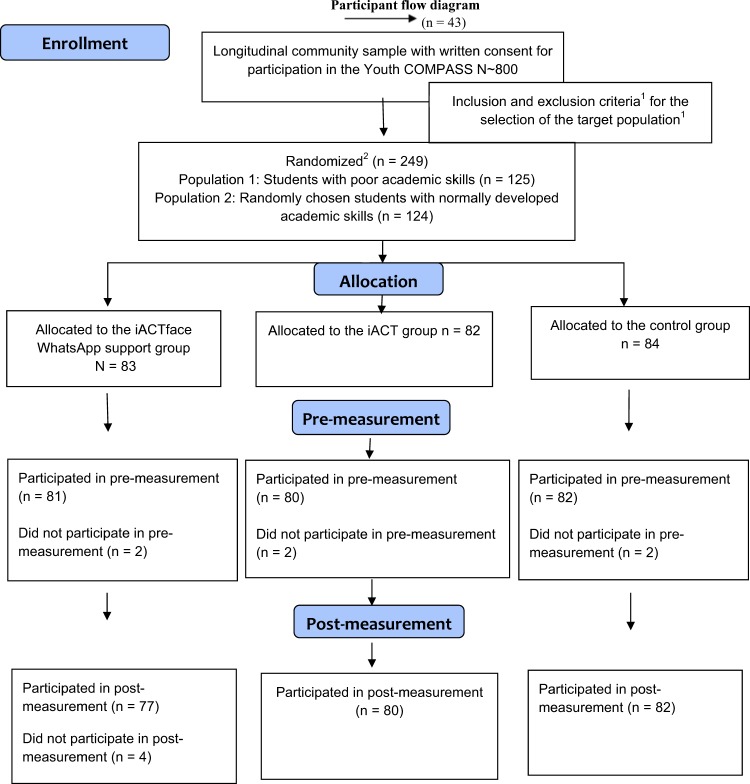
Flowchart. Note: The inclusion and exclusion criteria^1^ for the target population were as follows: Belonged to the larger longitudinal study basic group; had written consent for participating in the intervention; was a native Finnish speaker; and had previous data concerning reading and math skills and achievement scores from grades 6 and 7. Randomization^2^ was conducted in two phases: First, an equal number of male and female adolescents with poor academic skills were identified. Second, an equal number of same-sex classmates who had normally developed academic skills were randomized into the study. All participants were randomly allocated into three study conditions by an independent researcher

The basic inclusion and exclusion criteria for study participation were set (see Fig. 1) before the selection and randomization. The selection of the target sample and its randomization into the intervention and control groups included two phases. *In the first phase*, two subsamples of adolescents from the larger basic sample (*n*~800) were selected. First, a group of adolescents with poor academic skills (n = 125) (i.e., students who performed below the 16th percentile in reading or math tests during grades 6 and 7 [see a description of these tests in the Measures section] or students who belonged to the lowest 16th percentile in general academic achievement in their grade point average at the end of grade 7) was identified (see also Table 1). This cut-off definition identifies the same participants as a categorization defined as “1 deviation below the mean based on the sample normal variation” (see, e.g., Landerl et al. 2009). Second, a similarly sized group of adolescents with no signs of poor academic skills (*n* = 124) was identified from the same classrooms as the participants with poor academic skills. *In the second phase*, the participants from these two groups (n = 249) were further randomly allocated into three study groups by an independent researcher: the *iACTface group*, receiving both face-to-face and online support; the *iACT group*, receiving only online support; and the *control group*, receiving no additional support. Six of the randomized adolescents withdrew from the intervention or they could not be reached before starting the program, and no data were available for them. Thus, the final sample of this study consisted of 243 adolescents, 161 of whom took part in the Youth Compass intervention: 81 adolescents participated in the iACTface group and 80 in the iACT group. The control group consisted of 82 adolescents. Pre-data were available from 243 and post-data from 239 adolescents (see Flowchart, Fig. 1). Written consent for participation was obtained from both the adolescents and their parents during the spring of 2017. The participants’ mean age at the beginning of the study was 15.27 years (*SD* = 0.39), with an almost equal number of boys (*n=* 124; 51%) as girls (*n=* 119; 49%). Demographic and sample characteristics at the baseline are provided in Table 2.

**Table 1:** Participants in the different groups and analyses

Initial phase characteristics	iACTface group (*n* = 81)	iACT group (*n=* 80)	Control group (*n=* 82)
P*articipants in the intention-to-treat analyses N* (%)			
Female	44 (54.3)	37 (46.3)	38 (46.3)
Male	37 (45.7)	43 (53.7)	44 (53.7)
*Poor academic skills N* (%)			
Normally developing academic skills	41 (50.6)	40 (50.0)	40 (48.8)
Poor academic skills	40 (49.4)	40 (50.0)	42 (51.2)
*Reason for poor academic skills N* (%)			
Unknown reason for poor academic skills	18 (22.2)	16 (20.0)	18 (22.0)
Reading problems	9 (11.1)	11 (13.8)	11 (13.4)
Math problems	7 (8.6)	7 (8.8)	8 (9.8)
Both reading and math problems	6 (7.4)	6 (7.5)	5 (6.1)
*Participants in the per-protocol analyses N (% from the intention-to-treat protocol)*			
	64 (79.0)	58 (72.5)	82 (100)
*Included/excluded cases (N) in the per-protocol analyses*			
Female	39/5	33/4	38/0
Male	25/12	27/17	4/0

Two different analytical protocols (intention-to-treat and per-protocol) were used, as recommended for intervention studies (Ranganathan et al. 2016)

**Table 2:** Sample characteristics in the three groups

Baseline characteristics	All (*n=* 243)	iACTface group (*n=* 81)	iACT group (*n=* 80)	Control group *n=* 82)
Age M (*SD*)	15.27 (*0.39*)	15.25 (*0.30*)	15.27 (*0.33*)	15.29 (*0.50*)
*Gender*				
Female	124 (51%)	44 (54.3%)	37 (46.3%)	38 (46.3%)
Male	119 (49%)	37 (45.7%)	43 (53.8%)	44 (53.7%)
*Mother tongue*				
Finnish	230 (94.7%)	77 (95.1%)	74 (92.5%)	79 (96.3%)
Other than Finnish	8 (3.3%)	3 (3.7%)	3 (3.8%)	2 (2.4%)
Bilingual (Finnish + some other language)	4 (1.6%)	1 (1.2%)	2 (2.5%)	1 (1.2%)
*Living with*				
Mother and father	167 (68.7%)	52 (64.2%)	59 (73.8%)	56 (68.3%)
Only with mother or father	20 (8.2%)	12 (14.8%)	4 (4.1%)	4 (4.9%)
Alternately with mother and father	38 (15.6%)	11 (13.6%)	12 (15.0%)	15 (18.3%)
Others^a^	14 (5.7%)	4 (4.9%)	3 (3.8%)	7 (8.5%)
*Parental Education (primary caregiver)*				
A/B/C (%)^b^		33/25/42 (%)	34/20/46 (%)	41/22/37 (%)
missing cases^c^		21	9	14

^a^Living with mother and stepfather, father and stepmother, foster care or approved home. Parental education level: ^b^A = vocational upper secondary education or lower, B = vocational college degree, C = Bachelor’s degree or higher. Information of education level was missing^c^ in some cases

### Coaches

In accordance with previous study recommendations (Andersson and Titov 2014; Wozney et al. 2017) and experiences in web-delivered interventions (e.g., Lappalainen et al. 2015; Räsänen et al. 2016), the participants received coaching and support from 31 acceptance and commitment approach -trained undergraduate psychology students (83% women). The coaches were students at the bachelor’s (41%) or master’s (59%) level. They received a total of 18 h of acceptance and commitment therapy training and had access to weekly supervision by a licensed psychologist during the intervention, four hours in total, plus an extra two hours if needed. Half (48%) of the coaches reported that they had little prior experience of using acceptance and commitment programs, whereas 52% reported that they regularly used acceptance and commitment exercises in their studies and daily life. The coaches conducted the preliminary and post-interviews (with the iACTface group), introduced the participants to the intervention program and procedure, and remained in weekly contact with them (both the iACTface and iACT groups). Each coach was assigned three to ten participants to follow. They also participated in the pre- and post-measurements (for all three groups).

### The Group Protocols and Measurement Procedures

#### The iACTface group

The participants in the iACTface group received a web- and mobile-delivered intervention program called Youth COMPASS. Before the program, this group also had a face-to-face meeting with their individually assigned coach, which comprised a structured interview and discussion (45 min) about the adolescents’ current life situation. The interview questions were shortened and adapted for the adolescents from a psychosocial interview template (Strosahl et al. 2012). These adolescents were also given oral instructions and an instructions sheet with credentials for the Youth COMPASS, which explained how to work in the web program and how and when to complete the weekly assignments. In a second face-to-face meeting, which followed the five-week intervention, the adolescents in this group were interviewed by the coach about their intervention experiences. During the five-week intervention, these adolescents had a short weekly contact with their coach via instant text messages (using the WhatsApp mobile application. See https://www.whatsapp.com/).

#### The iACT group

The participants in the iACT group received the same web- and mobile-delivered intervention program as those in the iACTface group. However, the iACT group participants had no individual face-to-face meetings with their coach. Instead, they received credentials and a brief introduction to the Youth COMPASS program, instructions and an instructions sheet for the web program, and a timetable for weekly assignments. The iACT group participants also had short weekly contact with their coach via instant text messages (SMS).

#### The control group

The control group was not provided with intervention resources or feedback. They (as well as the iACTface and iACT groups) only received normal support from the school, such as the possibility to liaise with school health professionals regarding psychological and other well-being-related issues or to get personal support for learning difficulties.

### The Intervention Program

The Youth COMPASS is a five-week online program aimed at enhancing adolescents’ psychological flexibility by guiding them in exploring their interests, thoughts, emotions and sensations, setting goals, and changing behaviors according to their goals: learning acceptance, defusion, and mindfulness skills (week 1), broadening these skills into self-compassion (weeks 2–3), and learning adaptation skills for use in the adolescents’ personal and social life (weeks 4–5). The program was inspired by the acceptance and commitment intervention models for youth (e.g., Ciarrochiet al. 2012; Hayes and Chiarrochi 2015) and especially by the experiences gained by university students from the COMPASS program (Räsänen et al. 2016), although the youth version was designed to be more playful, and the exercises were shorter and modified into digital form. The program consisted of short texts, pictures, video clips, comic strips, and audio-based exercises and could be accessed via PC, laptop, tablet, or mobile phone. Each of the five modules was divided into an introduction and three different levels, including a set of short exercises based on the particular process of psychological flexibility. The participants had to complete at least two exercises in each level to be able to advance in the program. The first exercise was mandatory, whereas the other exercises could be chosen from a selection of four to seven short exercises. Thus, to complete a week of exercises, the participants needed to pass at least six different exercises, although it was possible to complete all the exercises if they so desired. Most of the exercises were offered in both written and audio-recorded form. Around half of the exercises demanded more mental orientation (such as the mindfulness exercises) and self-reflection, while the other half required behavioral responses (such as doing an exercise, writing a response, or seeking answers). Altogether, the intervention program included more than 90 exercises, which were usually no longer than five to 10 min.

The coaches were able to follow the progression of their personally guided adolescents and to provide motivational feedback via SMS once weekly using semi-structured questions. Before giving feedback, the coach was instructed to check via the program’s platform whether the adolescent had completed the weekly exercises. The weekly feedback via SMS comprised three semi-structured questions (shown in Table 3): *How are you doing? Please rate your mood during the last week on a scale from 4–10* (4 = very bad, 10 = very good). The scale from 4 to 10 is commonly used in Finnish schools for grading schoolwork; therefore, it was familiar for all of the participants. A question related to each step, such as *What is important to you? What could you do today or tomorrow to add joy and energy to your life? Do it!* (This is an example from feedback questions for Module 1). When the adolescent replied via SMS, the coach sent her/him an encouraging message with a closing phrase: *Looking forward to hearing from you next week!* If the adolescent did not reply, the coach was instructed to send an SMS as a reminder, wait one day, and re-send the SMS message with the feedback questions. If no answers were received, this procedure was repeated on the fifth day with a message saying that the coach would call the adolescent in a day or two. If no reply was received after these three consecutive SMS messages, the coach was instructed to call the adolescent. The structure and content of the Youth COMPASS intervention are detailed in Table 3.

**Table 3:** The structure and content of the youth COMPASS intervention and related coaching activities

Core modules	Aims	Levels (L1 to L3) and themes	Coach’s Questions^a^	Examples of exercises and activities
0. Introduction	Brief orientation to the online intervention	Getting started: Introduction (text & video)/Be Your Own Life’s Hero (3 videos)		A brief introduction to the Youth COMPASS program (worksheet)
1. Direction For Life ACT processes: Finding personal interests and goals	Recognition of activities that provide energy, well-being, and joy. Examining possible obstacles in achieving them. Taking actions and concrete steps toward personally valued goals.	Introduction L1: What is important in my life? L2: What do I want to achieve in my life? L3: What are the barriers to achieving goals?	What is important to you? What could you do today or tomorrow to add joy and energy in your life? Do it!	Text, video, and comic strip L1: Strength Cards L2: Goal Cards L3: BOLD^b^
2. Me And My Mind ACT-process: Promotingawareness of self and acceptance and cognitive defusion skills	Exploring automatic thoughts and feelings. Acceptance of thoughts, feelings, and memories as they are. Developing awareness of the self-as-context. Cognitive defusion, i.e., taking an observer’s perspective toward one’s own thoughts and feelings.	Introduction L1: Me and my mind L2: Watching one’s own mind L3: Practicing defusion skills	You can choose how to relate to your own thoughts. Try to act differently from what your mind suggests. See what happens. What could you do that provided you energy this week?	Video: Is The Mind So Clever? Video Thoughts About Myself L1: Thoughts As L2: Soap Bubbles L3: Thoughts In My Pocket
3. “Stalking Myself” ACT-process: Being in the present and practicing acceptance	Taking a new stance on my thoughts and feelings. Learning how to be mindful here and now and applying these skills to everyday life.	Introduction L1: Observing one’s senses L2: Being in this moment (NOW) L3: Applying skills to everyday life	Tell us what kinds of skills—related to being in the moment—you have used in your everyday life. What kinds of consequences did you observe while using them?	Video and comic strip: Feelings in My Body L1: Before You Snap Out L2: Mindfulness L3: Through Music
4. Me and Myself ACT-process: Recognizing the self as a context and providing self-compassion	Perceptions of oneself and learning to take a different perspective to one’s thoughts and emotions and applying these skills to one’s own life	Introduction L1: Who am I and how do I act L2: Changing one’s perspective L3: Getting rid of stories of oneself	Kindness toward self is important. How can you treat yourself kindly (in a similar manner as your friend who has difficulties)?	Text, video, and comic strip L1:You in Social Media L2: Sky and Weather L3: Me and My Blunders
5. Me and Other People ACT-process: Applying important actions to social life and being compassionate toward others	Promoting good relationships with friends and other people and applying ACT-based skills to social life.	Introduction L1: Being a friend to yourself and others L2: Living in the world L3: Facing challenging life situations	What kinds of good things have you done for other people in the past week, or what could you do next week?	Text, video, and comic strip L1: Critique Hurts L2: Change the World L3: Preparing For Stressful Events

Coach’s Questions^a^: All weekly contacts were started using two basic questions: (1) How are you doing? (2) Please rate your mood over the last week on a scale from 4–10. These were followed by the third question presented above. ^b^BOLD is an exercise by Ciarrochi et al. (2012)

### Measures

#### Measures for assessing academic skills

The students’ *academic skills*—*reading fluency, math skills, and general academic achievement*—were measured at earlier stages (grades 6 and 7) as part of the broader longitudinal study. This knowledge was utilized in the selection and randomization procedure (see study design and randomization) as well as in the analysis to examine whether the students’ academic skill levels influenced the intervention results.

#### Reading fluency

Reading fluency was measured with three tests. Two word reading tests – the Word Identification and Spelling Errors tests (Holopainen et al. 2004; Kiuru et al. 2011)—and one sentence reading test – the Salzburg Reading Fluency Test (Landerl et al. 1997; translated into Finnish by Sini Huemer)—were used.

In the first reading fluency task, *the Spelling Errors test*, the students were instructed to search for spelling errors in 100 words; the time limit for the whole task was 3.5 min. Each word included one error (an incorrect, extra, or missing letter), which the students had to mark by drawing a vertical line (for example, *carot=car|ot*). The students received one point for each correct line (maximum score 100). According to the manual (Holopainen et al. 2004), the test–retest reliability of this task was 0.83–0.86.

The second reading fluency task, the *Word Identification test*, contained 25 word chains, each with four different words written without spaces between them (e.g., *vaatturimustikkavalmishevonen* [tailorbilberryreadyhorse]). The students were instructed to draw an upright line between the end and beginning of each identified word as fast and as accurately as they could (e.g., *vaatturi*|*mustikka*|*valmis*|*hevonen*). The students received one point for each correctly drawn line within the time limit of 1.5 min, the maximum number of points being 100. According to the manual (Holopainen et al. 2004), the test–retest reliability of this task was high: 0.70–0.84.

Third, in the short version of the *Salzburg Reading Fluency Test*, the students were asked to read 36 sentences one by one and to mark whether the meaning of each sentence was true or false. This test is constructed in such a way that the sentences are easy to understand so as to capture reading fluency rather than reading comprehension. A time limit of 1.5 min was used instead of the 3.5 min used in the original test. The students received one point for each correct answer, with a maximum possible score of 36. According to the test manual, the reliability of the original Salzburg Reading Fluency Test was .95 for second-grade students and 0.87 for eighth-grade students. Cronbach’s alpha reliability for the arithmetic mean across the standardized scores in the three reading tests were .87 in grade 6 (fall) and 0.89 in grade 7 (spring).

#### Math skills

Math skills were assessed with the *Basic Arithmetic Test (*Aunola and Räsänen 2007; see also Räsänen et al. 2009) in grades 6 (fall semester) and 7 (spring semester). The test contained tasks in addition, subtraction, multiplication, and division. The students were asked to do mental calculations and to write their answers on the test paper. The test consisted of 28 tasks (e.g., 527 + 31 = ?; 15 –? = 9; 12 × 28 = ?), starting with easier tasks and getting progressively more difficult. The time limit for completing the test was three min. The students received one point for each correct answer, with a maximum possible score of 28. Cronbach’s alpha reliability was 0.82 in grade 6 and 0.85 in grade 7.

#### Measures used to evaluate the intervention results

Prior to the start of the intervention, the adolescents participating in the randomized controlled trial (the iACTface, iACT, and control groups) were called to a group-administered assessment in the classrooms during regular school hours. Pre-measurements were conducted in September to October 2017 and post-measurements in October to November 2017. The interval between the first and second measurements was seven weeks. The pre-measurements were accomplished before the start of the intervention, while the post-assessment questionnaires were administered six weeks from the beginning of the intervention. The questionnaires were administered to student groups in their classrooms during regular school hours. The primary focus in the current study was on *experienced overall stress, school stress, and academic buoyancy*, which were measured both in the pre- and post-assessments. *Adherence* to the intervention program was assessed in the post-measurement phase.

#### Overall stress

Overall stress was first explained to the participants in written form: “Stress refers to a situation where people feel tensed, restless, nervous, or anxious and have difficulties sleeping due to the things wandering in their mind.” Using a six-point scale (1 = Not at all, 2 = Only a little, 3 = To some extent, 4 = Quite much, 5 = A lot, and 6 = Very much; for the validity of the stress measure, see Elo et al. 2003), the participants were asked to answer “Do you feel this kind of stress at the moment?” Cronbach’s alpha between the pre- and post-phase measurements was 0.79. In a broader community sample of Finnish ninth-grade adolescents (*N* = 879), the mean of overall stress was 3.06, with a standard deviation of 1.4. These descriptive statistics form an age-level comparison point for the level of overall stress in the present sample.

#### School stress

School Stress was measured using a scale adapted from t*he Health Behavior in School-Aged Children (HBSC) study* (Currie et al. 2012, see also Kämppi et al. 2012) conducted by the World Health Organization. It comprises four items—(“I have too much schoolwork”; “Schoolwork is difficult for me”; “School work is tiresome”; “School-related things bother me even in my free time”)—assessed on a five-point Likert scale (1 = Completely disagree; 5 = Completely agree). Composite scores for school-related stress were created by calculating the mean of the students’ responses to the four items. Cronbach’s alpha reliabilities were 0.77 for the pre- and 0.80 for the post-measurements, respectively.

#### Academic buoyancy

*The Academic Buoyancy Scale* (Martin and Marsh 2008) comprises four items (“I don’t let study stress get on top of me”; “I’m good at dealing with school work pressures”; “I don’t let bad marks affect my confidence”; “I’m good at dealing with setbacks at school, e.g. negative feedback on my work or poor results”), which were assessed on a five-point Likert scale (1 = Completely disagree; 5 = Completely agree). Composite scores for academic buoyancy were created by calculating the mean of the students’ responses to the four items. Cronbach’s alphas for the scale were 0.87 for the pre- and 0.89 for the post-measurement phases of the study. In a broader community sample of Finnish ninth-grade adolescents (*N* = 879), the mean of academic buoyancy was 3.55 with a standard deviation of 0.89. These descriptive statistics form an age-level comparison point for the level of overall stress in the present sample.

#### Adherence to the intervention

To assess adherence to the intervention, the number of tasks fulfilled in the program were measured by asking the adolescents’ individually assigned coaches to circle the best-suited option from the following alternatives: 1 = student did not do any of the tasks, 2 = student did some tasks from the first one or two modules, 3 = student did tasks from three or four modules, 4 = student did at least two tasks from all the 5 modules, and 5 = student did most or all the tasks from the program. *The adherence criterion* (i.e., criterion for the acceptable number of exercises and participation in the intervention) was that the participant fulfilled tasks from at least three modules (scored > 3 in the adherence scale above). The students who met the adherence criterion used the Youth COMPASS program, on average, 6.62 different days (SD = 2.99 days) over the 5-week intervention. They were also generally relatively satisfied with the intervention (M = 7.83, SD = 1.15, range of scale = 4–10).

### Statistical Analysis

The main intervention results are reported using two analytical options: intention-to-treat and per-protocol (Ranganathan et al. 2016). First, the analyses explored the background of the study samples: success of randomization (i.e., whether the three randomized groups were similar at the initial stage of the study); adherence and attrition of the participants, analyzing whether the adolescents who did only a few or none of the intervention tasks from the first module differed from those who successfully committed to the intervention (i.e., fulfilled the adherence criterion). Second, the analyses explored the group differences: the results of the intention-to-treat analyses (i.e., differences in the initial level and changes in overall stress, school stress, and academic buoyancy in the three study groups using the whole randomized sample, *n=* 243); the results of the per-protocol analyses (i.e., the within- and between-group differences as well as effect sizes among those participants who fulfilled the adherence criterion). Finally, the analyses explored the possible influencing (moderation) effects of baseline stress and buoyancy levels and poor academic skills on the outcomes of the intervention.

Statistical analyses were conducted using Mplus (version 7, Muthén and Muthén 1998) and IBM SPSS Statistics 24. All analyses included the selected and randomized 243 participants, with only few missing values. The baseline differences in the demographic measures in the three groups were explored with generalized linear modelling tests and chi-square tests. The differences in the initial level and in the changes from the pre- to the post-assessment in the different groups were analyzed using hierarchical linear modelling (HLM) with full-information maximum likelihood estimation. HLM accounts for missing values at random and includes all available data.

Effect sizes (ES) were reported using Cohen’s d and were calculated as follows: First, the within-group ES was calculated by subtracting the mean scores of the pre-measurement from the mean scores of the post-measurement and dividing the score by the pooled standard deviation of the two conditions. Second, the corrected between-group ES were calculated by subtracting group difference in the pre-measurement phase from group difference in the post- measurement phase and dividing it by the pooled standard deviation of the pre- measurement phase. A between-group ES of 0.20 was considered small, 0.50 moderate, and above 0.80 large (Cohen 1988). In the power analyses conducted before the study, it was estimated that 20–30 participants per group would be the minimum sample size necessary to observe moderate-level ES values (d, 95% CI = .50–.80).

## Results

### Background of the Study Samples

#### Success of randomization

In the first phase, MANOVA tests were run for the continuous variables (overall stress, school stress, academic buoyancy, and age at pre-measurement) and chi-square tests for the categorical variables (gender and academic skills status) to examine whether the three groups differed from each other in the initial stage of the study. No significant group differences were found (see Table 1), indicating that the randomization was successful.

#### Adherence and attrition

All the participants (*n=* 243) were included in the intention-to-treat protocol analyses. The per-protocol analyses included the participants who fulfilled the adherence criterion. A total of 64 (79%) and 58 (73%) participants in the iACTface and iACT groups, respectively, met the criterion (see Table 1 for the participants in the different groups and different analyses).

Further analyses explored possible differences between the participants who were left out of the per-protocol analyses and those who were accepted into it. The group differences were studied based on the initial pre-measurement scores in overall stress, school stress, academic buoyancy, gender, and academic skills. The results indicated that the participants who were left out reported lower stress (*M* = 2.43, SD = 1.4, *n* = 35) than those who continued with the study (*M* = 2.98, SD = 1.4, *n* = 204 (2-tailed t-test: t(237) = 2.23; *p* = 0.028). The participants who dropped out of the study were mostly male (2-tailed t-test: t(55.7) = 3.34, *p* = 0.001). No other differences were observed.

#### Gender differences

In both the intervention groups, a remarkable number of male participants did not fulfill the adherence criterion for the per-protocol analyses: 32% of the participants in the iACTface group and 40% in the iACT group. In addition, in the sample for the per-protocol procedure, gender differences were observed in the initial level of overall stress (Girls: *M* = 3.39, SD = 1.37, *n* = 109; Boys: *M* = 2.51, SD = 1.21, *n* = 95; 2-tailed t-test: t(202) = 4.86, *p* = 0.000) and academic buoyancy (Girls: *M* = 3.29, SD = 0.80, *n* = 109; Boys: *M* = 3.88, SD = 0.73, *n* = 96; 2-tailed *t*-test: t(203) = 5.48, *p* = 0.000), but not in school stress or academic skills. The possible effects of gender were minimized using gender as a covariate in the per-protocol analyses.

### Differences in the Groups and Their Outcomes

#### Intention-to-treat analyses

In this phase, group differences in the initial level and changes in the outcome measures (overall stress, school stress, and academic buoyancy) were examined using the whole randomized sample. Two different analyses were conducted, in which the intervention groups (i.e. iACTface and iACT) were contrasted with the control group (intention-to-treat analyses, *n=* 243 in the three groups), and gender was used as a covariate. The Mplus analyses and Wald test showed no statistically significant changes *(p* > 0.05) in any of the outcome measures: The two different intervention groups did not differ from the control group in their changes during the intervention. Moreover, no differences were found in the changes between the male and female participants in these groups, although there were gender differences in the initial levels of stress and buoyancy in all the analyzed groups (*p<* 0.001).

#### Per-protocol-analyses

The per-protocol analyses were conducted for the subsample of participants, that is, those who successfully advanced in the program. In this phase, however, the iACTface and iACT groups were combined into one intervention group (*n=* 123), whose stress, school-related stress, and academic buoyancy scores were contrasted with those of the control group (*n* = 82). Gender was used as a covariate in the analyses. The Wald test (one tailed) showed a statistically significant decrease in overall stress (*p* = 0.037) and an increase in academic buoyancy (*p* = 0.013) among all participants, but no clear indication of changes in school stress, although a tendency toward it could be observed (*p* = 0.057) (see Table 4) among male participants whereas female participants this tendency was not observed. The between-group effect sizes were small (Cohen’s *d=* 0.22 for stress, *d=* 0.18 for school stress, and *d=* 0.27 for buoyancy), as were the within-group effect sizes (see Table 5).

**Table 4:** Mean scores and standard deviations at pre- and post-measurement in the intervention and control groups

Scale	Group	Pre-M (SD)	Post-M (SD)	Post- and Pre-Wald Test change estimate (df = 1), One-tailed *p*-values.
Overall Stress	INTERVENTION	3.10 (1.45)	2.91 (1.23)	3.19
	CONTROL	2.80 (1.22)	2.91 (1.33)	*p* = 0.037*
School Stress	INTERVENTION	2.84 (0.81)	2.84 (0.77)	2.49
	CONTROL	3.00 (0.83)	2.85 (0.92)	*p=* 0.057
Academic Buoyancy	INTERVENTION	3.46 (0.83)	3.69 (0.75)	4.91
	CONTROL	3.73 (0.78)	3.74 (0.82)	*p* = 0.013*

In the INTERVENTION group, the participants from the iACTface and iACT groups (*n* = 123) were combined. CONTROL group *n* = 82

* = *p* < 0.05

**Table 5:** Between-group and within-groups effect sizes (Cohen’s *d*, corrected)

Scale	Between pre-post (corrected Cohen’s d)	Within INTERVENTION Pre–Post	Within CONTROL Pre–Post
Overall Stress	0.22*	0.14	0.08
School Stress	0.18	0.01	0.17
Academic Buoyancy	0.27*	0.28*	0.01

In the INTERVENTION group, the participants from the iACTface and iACT groups (*n* = 123) were combined. CONTROL group *n* = 82

* = *p* < .05

### Moderation Effects

#### Influence of the initial levels of stress and buoyancy

Additional analyses were conducted to explore whether the initial levels of stress and academic buoyancy affected the results (using the sample selected for the per-protocol analyses, *n=* 123). Based on earlier intervention studies (Swain et al. 2015), it is possible that adolescents, who have higher stress and lower buoyancy levels, would benefit more from the intervention than those who have lower stress and higher buoyancy levels at the initial phase of the study. The results of the Wald test (one-tailed) and the moderation analyses conducted in MPLUS confirmed the results partly and showed that those who had higher baseline overall stress (*p* < 0.001) and higher baseline school stress (*p* < . 05) had greater positive gains than those who had a lower level of stress in the baseline state. In turn, the gains in academic buoyancy were not connected to the initial level of buoyancy (*p* > 0.05). All analyses were conducted using gender as a covariate.

#### Influence of academic skills

In the final step, SPSS analyses were conducted (using the sample selected into the per-protocol analyses, *n=* 205) to explore whether the adolescents with poor academic skills (*n=* 100) experienced a higher level of overall stress and school stress and a lower level of academic buoyancy compared to the participants with no signs of poor academic skills (*n=* 105). Finally, Mplus analyses were conducted to explore whether a poor academic skills status moderated the changes in the intervention outcomes. The analyses were conducted using separate intervention groups and by combining them into one intervention group, which were contrasted with the control group. The results showed that the students with poor academic skills did not differ from those with normally developed academic skills and who experienced changes in overall stress, school stress, and academic buoyancy (all *p*-values > 0.05).

## Discussion

Recent studies have shown that over 30% of adolescents in the West suffer from stress and related symptoms, which may have long-lasting effects on their subsequent health development and educational careers. On the other hand, individual factors, such as academic buoyancy, are proposed to protect youth from the effects of stress. The interesting question is whether modern brief therapeutic methods, such as the acceptance and commitment intervention model, could be modified and used to alleviate stress and to promote academic buoyancy in the school context. In the current randomized trial study, this issue was explored in a school setting using a general (non-clinical) sample of 249 adolescents, who were randomized into two intervention groups and a control group. The aim of the present study was to investigate the efficacy of a novel five-week web-based acceptance and commitment intervention program called Youth COMPASS in reducing adolescents’ stress and promoting their academic buoyancy.

All participants in the Youth COMPASS intervention performed short online tasks following the principles of acceptance and commitment approach and had weekly contact with a personal coach. Half of the adolescents in the Youth COMPASS intervention group also had two one-hour face-to-face meetings with their personal coach. The study also explored whether the two intervention groups differed from each other and whether the gains in the intervention groups were larger than in the control group. In addition, the study explored whether the adolescents’ poor academic skills influenced the intervention results. The results indicated that the two intervention groups did not differ from each other in terms of gains during the intervention, and in the following analyses, they were combined. When the total number of participants (*n=* 243; intention-to-treat analyses) were included in the analyses, no differences between the two intervention groups and the control group were found. However, when those participants who had fulfilled an acceptable number of tasks (at least three of the five intervention modules; *n=* 205; per-protocol analyses) were explored, statistically significant changes that were in line with the expectations were observed in overall stress and academic buoyancy in favor of the intervention groups. Additional analyses also showed that those who had higher stress in the initial stage of the study had greater positive gains in interventions than those who had low initial stress levels. Poor academic skills were found to have no effect on the intervention results.

The main aim of the current study was to explore the acceptance and commitment intervention effects on overall and school stress. In line with the expectations, during the 5-week intervention period, a small but significant decrease was observed in the level of overall stress in the intervention group but not in the control group. A similar trend regarding a group-level change was found in school stress, though it was marginally statistically significant. Burckhardt et al. (2016) conducted an acceptance and commitment approach and positive psychology-based intervention in an Australian school setting and showed a somewhat greater change in stress (as well as in depression and anxiety). However, their results varied considerably based on the grade level of the participants. Further, in the last grade of high school (a grade level comparable with that of the current sample), the effect sizes were even smaller than in the current study. Livheim et al. (2015) conducted a six-week acceptance and commitment based group session intervention aimed at decreasing stress among a small group of Swedish youth. In their study, the participants showed an impressive reduction in stress (*d=* 1.20 using the Perceived Stress Scale), which is in line with Livheim’s (2004) earlier intervention study. Thus, the results of the current novel intervention are in line with earlier studies using normal school samples, but they fall behind the gains in studies using clinical samples.

This article also explored whether the intervention influenced the adolescents’ academic buoyancy. This is likely the first acceptance and commitment intervention study in which academic buoyancy has been measured among adolescents. Buoyancy refers to one’s capacity to overcome everyday academic life setbacks and challenges successfully (Martin and Marsh 2009), and it can be assumed to be a kind of counterforce against stress. In line with the expectations, the current study showed a small but significant increase in academic buoyancy in favor of the two intervention groups. The magnitude of the results is comparable, though contrary, to the changes in overall stress in the current study. The current findings support the theoretical views of acceptance and commitment and its assumed ability to influence multiple core psychological skills (Dindo et al. 2017) and, via them, coping skills, such as academic buoyancy. In other words, the results of the current study propose that intervention increases self-awareness, acceptance, and defusion skills in relation to challenging situations in academic life. It likely also promotes the ability to set personal goals, as well as the courage to take independent actions in one’s life. These, in turn, will enhance academic buoyancy.

The results of the current study are in line with the promising results of some previous face-to-face interventions (e.g., Burckhardt et al. 2016; Livheim et al. 2015), as well as theoretical views on interventions involving adolescents (Ciarrochi et al. 2012; Hayes and Chiarrochi 2015). They also corroborate findings concerning the possibilities of web and phone technology possibilities in interventions (Andersson and Titov 2014; Wozney et al. 2017) and recent empirical explorations of these possibilities among adult participants (e.g., Kinnunen et al. 2018; Lappalainen et al. 2014a; Lappalainen et al. 2015; Puolakanaho et al. 2018; Räsänen et al. 2016). The results of the current article concerning overall stress and academic buoyancy also support the view and results of earlier studies showing that acceptance and commitment programs may have an effect on deep psychological skills and may, therefore, influence different psychological well-being and health-related factors (Dindo et al. 2017; Hayes and Hofmann 2017).

The current article also explored whether poor academic skills moderated the effects of the intervention. The results indicate that poor academic skills had no effect on the efficacy of the intervention, suggesting that the intervention was equally effective for students with poor academic skills as it was for those with normally developed skills, which is a novel finding in the field of acceptance and commitment approach. In addition, neither adherence nor commitment to the intervention depended on the students’ academic skills. In the current intervention program, possible problems resulting from poor academic skills were considered during the planning of the program; therefore, most of the exercises were available in both text and audiovisual formats. The finding suggests that regardless of the level of academic performance, every student can benefit from the program. Given the exceptionally high prevalence of poor academic skills among the student population (Cortiella and Horowitz 2014), this finding is important, as it suggests that the usability and efficacy of this kind of intervention does not depend on the level of academic skills. The finding is also in accordance with theoretical views on acceptance and commitment therapy approach and the supposed change mechanism (e.g., Hayes et al. 2012), which proposes that academic skills are not connected to changes in core acceptance and commitment processes.

Notably, in the current research, the majority of the participants did not have clinically and personally significant symptoms, such as stress or anxiety, nor did they undergo corresponding treatment; therefore, there was no personally driven motivation to participate in the intervention program, as in most prior acceptance and commitment studies. It is noteworthy that earlier studies have indicated that effects from interventions among general samples are usually smaller than among clinical samples (Swain et al. 2015). It is notable that the gains in the current intervention were nevertheless greater among those who had a higher level of overall or school stress than those who had a lower level of stress in the initial phase of the study. In this light, the small intervention-related changes found in the current study seem more promising. There may also be other explanations for the results; for example, the measures (scales), despite showing good internal consistency, may not be sufficiently sensitive to tap changes.

Although the participants’ commitment and motivation toward the program were supported in several ways—for example, via weekly contact with the coach, the structured content of the intervention program, and, in many cases, the game-like exercises designed specifically to appeal to adolescents—it must be borne in mind that adherence to the intervention was not optimally shown for all adolescents. In total, 25% of the adolescents did not meet the adherence criteria, with a relatively higher number of male versus female participants lacking adherence to the program. One reason for the lower adherence among some adolescents is that the participants were expected to do the exercises in their own leisure time, as the program was not part of the regular school curriculum. However, it is still notable that the majority, that is, 75% of the adolescents, did finish the program. Considering this and the fact that the intervention demonstrated clear positive effects, the results of the current study are promising.

The current study introduced a new mobile- and web-delivered acceptance and commitment program called Youth COMPASS, which was used with the aid of close or distant personal contact with a coach. The findings suggest that support that is more distant worked as well as the model with closer personal contact. This is in line with the recent results of Lappalainen et al. (2014a, 2014b), which suggest that web-delivered acceptance and commitment interventions without personal meetings may work better than the same intervention involving personal meetings with a coach. These findings may imply that technology-based interventions may include additional elements that further support the development of independency and self-knowledge skills (such as awareness, acceptance, defusion skills, and valued actions) related to acceptance and commitment therapy. In other words, the results suggest that technology-based features could further promote the targeted goals of acceptance and commitment intervention.

### Limitations and Future Directions

In the present study, no differences were found in the intervention outcomes between the two intervention models, the web- and mobile-delivered acceptance and commitment intervention model, which included an extra hour of personal contact at the beginning and end of the study, and the model without personal face-to-face contact. The results are promising, as they suggest that the web-based intervention was equally effective with or without extra face-to-face contact in a general (non-clinical) sample. However, in future research, it would be interesting to explore the influence of a larger variation in face-to-face contact on the intervention results, together with varying levels of initial symptoms. The current study can be considered a pilot study exploring the possibilities of a mobile-delivered brief acceptance and commitment intervention for preventive purposes among youth. In future studies and in the implementation of the interventions into clinical or school practice, it is important to involve parents, teachers and other important figures in adolescents’ lives in the intervention in the early phases of its planning.

When attempting to generalize the findings, it is worth noting that the participants were not selected from a clinical sample but rather randomly selected from a general student population, with an emphasis on poor academic skills. In other words, the gains in the current article are based on average changes in a group of a general (non-clinical) sample of adolescents. When considering the clinical meaningfulness of the findings, one must consider the sensitivity of the measures, the severity of the symptoms (i.e., the amount of stress and academic buoyancy), and their prevalence in the explored sample. This issue can be estimated using statistical tools if the effect size and prevalence of disorders in a specific sample are known (see the illustrative presentation by Coe 2002; see also Griner et al. 1981). Following this, the effect sizes found in the present study (although being in line with those in general samples; see Swain et al. 2015) were weaker than typically observed among clinical samples, with specific difficulties in stress management and academic buoyancy (e.g. Livheim et al. 2015; Livheim 2004). Yet, the found effect sizes were also good in light of the statistical view presented above (i.e. Coe 2002 and Griner et al. 1981). However, in the future, clinical study samples should also be used to obtain insights into the clinical significance of the effects of the program.

Another limitation relates to the lower adherence rates among male participants compared to those among females. An analysis of the non-completers (25% of adolescents randomized to the intervention groups) showed that they were mostly male, and they reported having lower levels of initial stress and higher buoyancy than those who completed the program (see also APA 2014; Wiklund et al. 2012). However, in some earlier studies, boys have also been observed to use avoidance and distraction methods as a means for coping with stress (APA 2014; see also the Teen Help website). Thus, the high number of male participants who did not experience stress or commit to the program may reveal an avoidance and distraction strategy rather than actual stress levels and interest in the program. Nevertheless, it seems that male participants do not as easily commit to this kind of intervention as females. Motivation strategies, especially by male participants, ought to be carefully considered in future intervention studies. The focus of the current study was on 15- to 16-year-old ninth-grade adolescents who were attending their last year of lower secondary school. In future studies, it would be important to explore whether this kind of brief intervention is also useful among younger and older samples of youth, as well as in other educational and cultural contexts.

A final limitation is that only stress, school-related stress, and academic buoyancy were examined as outcomes of the intervention. Because acceptance and commitment practices are thought to have broad effects on well-being (e.g., Hayes et al. 2012), the reported measures give a slightly narrow view of the possible effects of the Youth COMPASS intervention. It is also notable that the two used measures of stress may tap different aspects of it and thereby influence the results. The overall stress measure is likely to assess more strongly the degree of current stress symptoms, whereas the school stress scale is likely to assess the long-term experiences of school-related stress (i.e., the extent to which school-related demands exceed students’ resources) that resemble students’ experienced level of school-related exhaustion (Salmela-Aro et al. 2009). These differences might have partly affected the measures’ sensitivity to detect short-term changes in the stress experiences (cf. relatively stronger intervention effects were observed overall stress than in relation to school-related stress). In addition, the role of the coach–participant interaction in motivation or the impact of the number and different types of intervention exercises on the outcomes could not be explored in the current article, but these will be important to clarify in future studies. More studies are needed to confirm the findings of the current study.

### Practical Implications

The findings of the current study are promising and propose that this kind of intervention could be used as a preventive and early tool for alleviating stress and promoting coping skills among adolescents. The study also suggests that it would be useful to investigate further the potential of web-based acceptance and commitment interventions among adolescents. This study opens possibilities to expand the repertoire of currently available school-based programs. It would be interesting to fit the program into regular school curriculum practices and to apply it to all pupils in a class. Web and mobile technology makes this kind of intervention feasible, easy to implement, and cost effective, and it may reduce the risk of stigma by normalizing interventions provided for mental health (Ciarrochi et al. 2012; Hayes and Ciarrochi 2015). In addition, the program could generate new ideas and understanding for teachers and other professionals in their work with adolescents.

An intervention study that applies Youth COMPASS to a clinical sample would be interesting given the findings of the current study. It is likely that the program would work even better with adolescents who experience psychological distress and who may be more highly motivated to participate in and take advantage of the program. However, when using clinical samples, it would be important to provide participants with the possibility of personal contact with a healthcare professional. In addition, small weekly group meetings could provide different therapeutic elements, such as peer support for same-age adolescents with similar experiences (Livheim 2004; Livheim et al. 2015). This could further help adolescents become more aware and accepting of their inner experiences and to achieve their personal life goals and interests, thereby promoting the targets of acceptance and commitment therapy (Ciarrochi et al. 2012; Hayes and Chiarrochi 2015).

Modern technology can also increase the possibilities of applying programs to diverse settings, such as schools and in a variety of leisure activities. These kinds of interventions do not necessarily demand specialized skills, and they can be used by different kinds of professionals working with adolescents. In addition, the programs can also increase adults’ understanding of psychological skills that are important not only for adolescents, but also for all human beings. However, it is also important to observe that the theoretical views that are mostly “hidden” in the program can be misunderstood, which may lead to misuse and a devaluing of the program if users do not have proper knowledge of the theoretical background behind the program. In addition, modifications are needed for future study designs and protocols.

## Conclusions

Acceptance and commitment interventions have been used thus far among adult samples with clinical disorders and symptoms, but little is known about how the interventions work as preventive and early tools in alleviating diverse psychological symptoms (here, stress) and promoting well-being and health (here, academic buoyancy) among non-clinical samples of adolescents. The current study presented a novel intervention, Youth COMPASS, aimed at promoting adolescents’ mental health by combining mobile and web technology, along with the acceptance and commitment model. The program includes multiple, brief exercises that have features resembling more games than traditional intervention exercises. Of interest was also the examination of whether academic skills affect the intervention results. The results demonstrated a statistically significant reduction in symptoms of overall stress and an increase in academic buoyancy. In addition, the gains in the interventions were larger among those whose stress levels at the initial stage of the study were highest. Moreover, poor academic skills did not influence the intervention outcomes. The results suggest that acceptance and commitment models and programs are also feasible for early intervention among young people. Furthermore, the results indicate that mobile technology may be of assistance in youth interventions and may provide elements—such as enhanced self-knowledge and more autonomy over one’s actions—that promote the targeted goals of acceptance and commitment therapy. This, in turn, opens new directions for enhancing the health and well-being of adolescents, which can be applied in diverse settings, including schools. More detailed studies and analyses of the contents of the program and their connection to the intervention gains are needed. Special attention is needed to build the motivational aspects of the exercises before and during the program.

## Appendix 1

Table 6

**Table 6:** Mean scores and standard deviations at pre- and post-measurement in intervention groups

Scale	Group	Pre-M (SD)	Post-M (SD)
Overall Stress	iACT	2.98 (1.33)	2.73 (1.18)
	iACTface	2.91 (1.53)	2.87 (1.30)
School Stress	iACT	2.93 (0.80)	2.88 (0.76)
	iACTface	2.88 (0.85)	2.85 (0.77)
Academic Buoyancy	iACT	3.53 (0.84)	3.75 (0.79)
	iACTface	3.54 (0.86)	3.70 (0.79)

In the iACT group (*n* = 80), iACTface (*n* = 81)
